# Metagenomic analysis of MWWTP effluent treated via solar photo-Fenton at neutral pH: Effects upon microbial community, priority pathogens, and antibiotic resistance genes

**DOI:** 10.1016/j.scitotenv.2021.149599

**Published:** 2021-12-20

**Authors:** Pâmela B. Vilela, Rondon P. Mendonça Neto, Maria Clara V.M. Starling, Alessandra da S. Martins, Giovanna F.F. Pires, Felipe A.R. Souza, Camila C. Amorim

**Affiliations:** a Universidade Federal de Minas Gerais, Escola de Engenharia, Departamento de Engenharia Sanitária e Ambiental, Research Group on Environmental Applications of Advanced Oxidation Processes (GruPOA), Av. Pres. Antônio Carlos, 6627, 31270-901 Belo Horizonte, MG, Brazil; b Universidade Federal de Minas Gerais, Instituto de Ciências Biológicas, Departamento de Bioquímica e Imunologia, Pampulha, Belo Horizonte, MG, Brazil

**Keywords:** Antimicrobial resistance, Tertiary municipal wastewater treatment, One Health, Whole Genome Sequencing, 16S rDNA sequencing, Bioinformatic analysis

## Abstract

The effectiveness of advanced technologies on eliminating antibiotic resistant bacteria (ARB) and resistance genes (ARGs) from wastewaters have been recently investigated. Solar photo-Fenton has been proven effective in combating ARB and ARGs from Municipal Wastewater Treatment Plant effluent (MWWTPE). However, most of these studies have relied solely on cultivable methods to assess ARB removal. This is the first study to investigate the effect of solar photo-Fenton upon ARB and ARGs in MWWTPE by high throughput metagenomic analysis (16S rDNA sequencing and Whole Genome Sequencing). Treatment efficiency upon priority pathogens and resistome profile were also investigated. Solar photo-Fenton (30 mg L^−1^ of Fe^2+^ intermittent additions and 50 mg L^−1^ of H_2_O_2_) reached 76–86% removal of main phyla present in MWWTPE. An increase in *Proteobacteria* abundance was observed after solar photo-Fenton and controls in which H_2_O_2_ was present as an oxidant (Fenton, H_2_O_2_ only, solar/H_2_O_2_). Hence, tolerance mechanisms presented by this group should be further assessed. Solar photo-Fenton achieved complete removal of high priority *Staphylococcus* and *Enterococcus*, as well as *Klebsiella pneumoniae* and *Pseudomonas aeruginosa*. Substantial reduction of intrinsically multi-drug resistant bacteria was detected. Solar photo-Fenton removed nearly 60% of ARGs associated with sulfonamides, macrolides, and tetracyclines, and complete removal of ARGs related to β-lactams and fluoroquinolones. These results indicate the potential of using solar-enhanced photo-Fenton to limit the spread of antimicrobial resistance, especially in developing tropical countries.

## Highlights

• Photo-Fenton had significant impact on secondary wastewater bacteria diversity.
• *Proteobacteria* enrichment in the presence of hydrogen peroxide must be investigated.
• Total removal of priority pathogens *K. pneumoniae* and *P. aeruginosa* by photo-Fenton
• Complete removal of resistance genes related to β-lactams and fluoroquinolones
• Sul1, tet(X), tet(C) are potential indicators of antibiotic resistance in wastewaters.

## Graphical abstract

**Figure:**
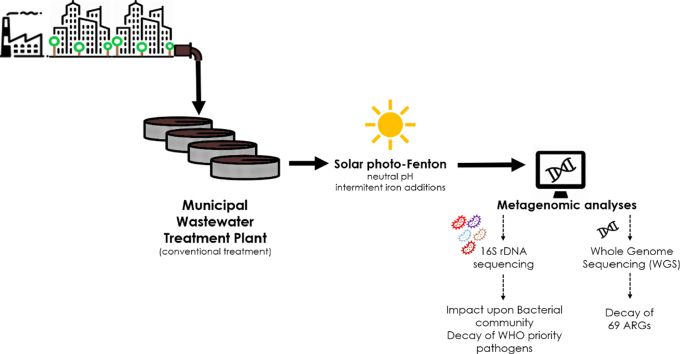

## 1 Introduction

Antimicrobial resistance (AMR) challenges the treatability of infectious diseases as it decreases the performance of antibiotics used to treat infected patients. Treatment processes applied in Municipal Wastewater Treatment Plants (MWWTP) play a key role on the spread of ARB and ARGs to the environment. The collection of ARGs present in MWWTPE (resistome) is influenced by the high density and rate of interaction between microbial communities aligned to subinhibitory concentrations of antibiotics in biological reactors. These factors favor ARG transfer to non-resistant strains resulting in ARB enrichment in the discharged effluent (Murray et al., 2018; Stanton et al., 2020). Therefore, reducing AMR in MWWTP remains a critical challenge (Fiorentino et al., 2019; Vikesland et al., 2019).

The World Health Organization (WHO) highlights that the surveillance of critical hotspots of AMR (i.e., MWWTP) is essential. In order to monitor the threat, it is necessary to take a “One Health” approach involving coherent and concerted multisectoral (human, animal, and environmental) actions to counter AMR at various levels. Hence, many countries adopt this perspective by tackling the spread of ARB promoted by MWWTPE discharge (Collignon and McEwen, 2019). The WHO prioritized ARB on their list of ‘global priority pathogens’ which pose the greatest threat to human health, such as some bacterial species and their accompanying resistome (collection of ARGs carried by these species) (e.g., carbapenem-resistant *Acinetobacter baumannii*; cephalosporin-resistant *Klebsiella pneumonia*; vancomycin-resistant *Enterococcus faecium*, etc.) (WHO, 2017; Starling et al., 2021b). Consequently, investments in the development of advanced wastewater treatment strategies that promote the removal of ARB and ARGs from municipal wastewater (MWW) prior to discharge have increased.

Many studies indicate that tertiary treatment technologies such as chlorination, UV-C, and ozonation are ineffective to remove ARB and ARGs from MWWTPE (Di Cesare et al., 2020; Lee et al., 2017; Narciso-da-Rocha et al., 2018). Chlorination may select ARB favoring their spread and affects intra and extracellular concentrations of ARGs (Guo et al., 2015; Hou et al., 2019; Liu et al., 2018). Recently, ozonation has been associated with the selection of *Pseudomonas aeruginosa* (Moreira et al., 2021), a priority pathogen according to the WHO (2017). In contrast, Advanced Oxidation Processes (AOP) are feasible methods for the inactivation of bacteria and elimination of ARGs as oxidative radicals damage cell membrane and DNA structure through free radical reactions (Guo et al., 2020; Li et al., 2021; Michael-Kordatou et al., 2018). Even though, regrowth was observed after treatment by some AOP treatments especially H_2_O_2_ + sunlight (Fiorentino et al., 2015; Michael et al., 2020; Wang et al., 2021).

Photo-Fenton (Fe^2+^ + H_2_O_2_ + UV–Vis) has been confirmed to promote effective removal of ARB and ARGs and to inactivate cell-free ARGs from MWWTPE (Michael-Kordatou et al., 2018; Vilela et al., 2021; Starling et al., 2021a). Since photo-Fenton may be carried out under sunlight, its investigation for the improvement of MWWTPE quality in areas of high solar irradiance (i.e., tropical developing countries) must be further stimulated. Yet, one of the main limitations of photo-Fenton treatment is the optimal pH of operation (2.8–3.0). Considering the natural pH of MWWTPE (6.0–7.5), different strategies have been investigated to apply photo-Fenton at a neutral pH level (Clarizia et al., 2017). A feasible alternative for this purpose is the intermittent iron addition strategy which assures the presence of soluble and reactive Fe^2+^ species throughout treatment (Carra et al., 2014, Carra et al., 2013; Díaz-Angulo et al., 2021). This strategy has been proven effective for disinfection and ARB removal (Starling et al., 2021a).

Nevertheless, the quantification of ARB and ARGs in MWWTPE samples and analysis of solar photo-Fenton impact upon resistome profile is still challenging. Most studies apply culture-dependent methods (Ioannou-Ttofa et al., 2019; Michael et al., 2020; Moreira et al., 2018; Rodríguez-Chueca et al., 2019; Starling et al., 2021a), which are relevant as they prove the viability of ARB and expression of ARGs after treatment. Yet, culture-dependent methods may be inadequate to analyze treatment effects upon uncultivable organisms, which represent public health risks (Manaia et al., 2018; Vaz-Moreira et al., 2011). In contrast, metagenomic analyses such as 16S rDNA sequencing show high specificity and sensitivity for all organisms, no matter their viability, and enable the analysis of treatment impact upon microbial community and resistome, which are fairly diverse in MWWTPE (Rizzo et al., 2013). In addition, Whole Genome Sequencing (WGS) enables the identification of all genes present in a sample using high throughput screening (Ishii, 2020; Rice et al., 2020). So far, no previous studies have assessed WGS profile of MWWTPE treated by solar photo-Fenton. Besides, only a few studies analyze treatment efficiency upon priority pathogens listed by the WHO (WHO, 2017) and present in MWWTPE.

The goal of this study was to investigate the effects of the solar photo-Fenton process upon priority pathogens, bacterial community, and ARGs present in MWWTPE by using metagenomic analyses (16S rDNA sequencing and WGS) with a deep examination of the effect of the proposed treatment upon WHO critical priority pathogens and resistome profiles.

## 2 Material and methods

### 2.1 Reagents

All reagents used in the experiments were of analytical grade. Hydrogen peroxide (H_2_O_2_, 35%) and sulfuric acid (H_2_SO_4_, 98%) were purchased from Neon. Heptahydrate ferrous sulfate (Fe·SO_4_·7H_2_O) and ammonium metavanadate (NH_4_VO_3_) were provided by Nuclear. Bovine Serum Catalase (H_2_O_2_:H_2_O_2_ oxidoreductase) and acetic acid (CH_3_COOH, 96%) were purchased from Sigma-Aldrich. 1,10-Phenanthroline (C_12_H_8_N_2_.H_2_O) were provided from Vetec. Synth supplied ammonium acetate (CH_3_COONH_4_). DNA Ladder was purchased from Promega.

### 2.2 MWWTPE sampling

MWWTPE was sampled in the output of a secondary settling tank from a conventional activated sludge system throughout a whole year, comprising wet and dry seasons. Physicochemical characterization of MWWTPE was performed for all samples, as presented in Table S1.

### 2.3 Solar photo-Fenton treatment

Solar photo-Fenton (Fe^2+^ + H_2_O_2_ + solar) treatment of MWWTPE was conducted in a solar simulator chamber (SUNTEST CPS+, ATLAS) containing a Xenon lamp (300–800 nm) set at 268 W m^−2^ (30 W m^−2^), corresponding to the annual average irradiance in Belo Horizonte/MG. Temperature was kept constant at 35 °C. Experiments were performed at neutral pH in a glass recipient (400 mL) under continuous stirring. Preliminary Fenton and solar photo-Fenton experiments (30 mg L^−1^ of Fe^2+^ and 50 mg L^−1^ of H_2_O_2_) were conducted for 120 and 240 min to determine the most appropriate reaction length. Then, reactions were performed with 5 mg L^−1^ and 30 mg L^−1^ Fe^2+^ (intermittent additions: 0 min = 15 mg L^−1^; 5, 10 and 15 min = 5 mg L^−1^) in the presence of 50 mg L^−1^ of H_2_O_2_ (240 min) to determine the most appropriate iron concentration. These reagent concentrations were defined according to Vilela et al. (2021). Accumulated irradiation (Q_uv_) during treatments was calculated as according to Malato et al. (2009).

Solar photo-Fenton was performed using 30 mg L^−1^ Fe^2+^ (intermittent additions) and 50 mg L^−1^ of H_2_O_2_ for 240 min at neutral pH in all subsequent treatments. Controls consisted of Fenton (Fe^2+^ + H_2_O_2_), Fe^2+^ alone, solar + Fe^2+^, H_2_O_2_, solar + H_2_O_2_ and solar irradiation alone under the same operational conditions. Samples were withdrawn during reactions for residual H_2_O_2_ (Nogueira et al., 2005) and Fe^2+^ quantification (APHA, 2017). DNA extraction for ARB and ARG analysis was carried out after 240 min of treatment (Q_uv_ = 22.28 kJ L^−1^). Catalase enzyme (460 mg L^−1^ in 0.04 M phosphate buffer) was added to consume residual H_2_O_2_ (Poole, 2004).

### 2.4 Culture-based analysis of antibiotic susceptibility for MIC

The determination of ARBs was performed by antibiotic susceptibility testing for minimum inhibitory concentrations (MIC) (CLSI, 2015). A non-selective agar medium was used to evaluate the growth of total heterotrophic bacteria (THB) present in MWWTPE before and after the proposed treatment. At the same time, ARB quantification was performed in agar medium enriched with sulfamethoxazole (SMX, 350 mg L^−1^), trimethoprim (TMP, 350 mg L^−1^), ciprofloxacin (CIP, 4 mg L^−1^), tetracycline (TET, 16 mg L^−1^), and amoxicillin (AMX, 32 mg L^−1^). All plates were incubated at 37 ± 1 °C for 48 h, and colony-forming units were quantified in each plate.

### 2.5 DNA extraction, quality control, library preparation, and sequencing

Total DNA extraction was performed using FastDNA® Spin Kit for Soil (MP Biomedicals). DNA concentration and purity were measured by a NanoDrop UV–Vis spectrophotometer (Thermo Fisher Scientific), and structural integrity was determined by 1% agarose gel electrophoresis. Extracted DNA was shipped to Macrogen for library preparation and sequencing. Paired-end fragment libraries with a length of 450 nt from the 16S rDNA V3-V4 region were constructed using the primers 338F ACTCCTACGGGAGGCAGCA and 806R GGACTACHVGGGTWTCTAAT. 300 nt reads of each end were sequenced from fragments (Illumina MiSeq platform). Entire genomic DNA libraries were produced and sequenced (Illumina HiSeq) in 150 nt paired ends reads for WGS.

### 2.6 Bioinformatics analysis

#### 2.6.1 Taxonomic assignment

All pre-processing was carried out using the Micca software (Albanese et al., 2015). Sequence read pairs were merged and quality filtering was performed by trimming primer adapters from the concatenated sequences and removing low-quality sequences (0.75% max. error and 400 nt min size). Operational Taxonomic Units (OTUs) were generated de novo by multiple and global alignments within each sample by grouping those which contained more than 97% identity. Next, resulting OTUs were classified taxonomically (Ribosomal Database Project) (Cole et al., 2014). The NAST algorithm globally aligned OTUs to generate phylogenetic profiles. Pre-processed data were used as input in R 3.6.3 (https://www.r-project.org/), Phyloseq (McMurdie and Holmes, 2013), and vegan (Oksanen et al., 2019) packages.

A total of 7,486,167 high-quality sequences (>465 bp) were retained by 16S sequencing analysis. Good's coverage was higher than 99% (Good, 1953), indicating that the dataset was representative of the bacterial communities present in samples. Relative abundance in MWWTPE samples was compared by Kruskal-Wallis and Wilcoxon tests (a = 0.05) (Segata et al., 2011). Diversity and richness were calculated using rarified and non-rarefied versions of bacterial counts (Rowe and Winn, 2018). Non-rarefied counts were used for further analysis to avoid false positives and data loss (McMurdie and Holmes, 2014). Diversity degrees were accessed by beta-diversity (PCoA) using the Bray-Curtis dissimilarity index. Alpha-diversity analyses (“Observed” index and non-parametric methods “Chao1” and “Ace”) were used for estimating the number of species (Kim et al., 2017). A phylogenetic tree was drawn (100 bootstraps) containing priority pathogens (WHO, 2017) and other relevant species (Machado et al., 2020). DESeq2 R packages were used to compare the abundance of classes present in MWWTPE and treated samples (Love et al., 2014).

#### 2.6.2 Identification of ARGs

Sequenced reads were checked for quality (FastQC) (Wingett and Andrews, 2018) and filtered by trimming primer adapters and low-quality sequences (Q < 30) using Trimmomatic (Bolger et al., 2014). Reads were mapped to the ARG reference database Resfinder (Bortolaia et al., 2020) by Groot software (Rowe and Winn, 2018). All sample reads were partially assembled in de Bruijn graphs MetaCherchant software (Olekhnovich et al., 2018) to correlate ARGs with host species by Kraken 2 (Wood et al., 2019) against the NR database (O'Leary et al., 2016). A heatmap with ARGs abundance, classes, and GC content was plotted using R package pheatmap (https://cran.r-project.org/web/packages/pheatmap/index.html) and Circos (Krzywinski et al., 2009). Inhouse perl and R scripts were used to parse data.

Analysis of genes which confer resistance to H_2_O_2_ (KatA1, KatA2, KatMn, and KatE, AhpCF, Gpx1, Gpx2, and Gpx3) were carried out using filtered reads aligned against catalase (E1S7Y1_HELP9, G0L8N0_ZOBGA and CATE_ECOLI), hydroperoxidase (Q9RQ72_BACFG), and glutathione peroxidase (GPX1_SYNY3, GPX2_SYNY3 and A0A5P9CBT8_9PSED) peptide sequences through tblastn aligner (Camacho et al., 2009). Iinhouse bash scripts were used to parse results and measure abundance.

## 3 Results and discussion

### 3.1 Bacterial community in MWWTPE

Phylogenetic analysis of MWWTPE bacterial community is summarized in Fig. S1. *Proteobacteria* was the dominant phylum in all samples (29.55% ± 10.87% of total sequences), followed by *Actinobacteria* or *Bacteroidetes*, for which occurrence varied seasonally. *Actinobacteria* (20.50 ± 10.78%) prevailed in samples from the dry season, whereas *Bacteroidetes* (19.70 ± 6.83%) were predominant in samples from the wet season. Phyla *Chloroflexi* (11.71 ± 9.68%), *Firmicutes* (3.08 ± 3.38%), and *Acidobacteria* (2.16 ± 1.88%) were also represented in samples. *Proteobacteria* along with divergent proportions of *Bacteroidetes*, *Chloroflexi*, *Actinobacteria*, *Acidobacteria*, and *Firmicutes* were also detected in MWWTPE from activated sludge reactors worldwide (de Celis et al., 2020; Numberger et al., 2019). No significant differences concerning bacterial composition were detected in the different samples (p < 0.05). This indicates the stability of MWWTPE microbial community and reflects the operational consistency of the activated sludge system applied in the MWWTP.

### 3.2 Effect of solar photo-Fenton on bacteria community

Solar photo-Fenton reaction applied for 240 min (22.28 kJ L^−1^, 49.5 mg L^−1^ of H_2_O_2_ consumption) was the most efficient condition for the reduction of microbial community diversity (Fig. S2c). In solar processes, treatment efficiency is highly associated with accumulated irradiation during treatment (240 min = 22.28 kJ L^−1^; 120 min = 11.14 kJ L^−1^). The incident irradiation used in this study was equivalent to 30 W m^−2^ which equals average incident irradiation in tropical locations. In this way, reaction time and reactor volume for the application of solar processes must be determined for each location and season after the conduction of scale-up experiments (Starling et al., 2021b). Enhanced disinfection rates are expected to occur after prolonged exposure to irradiation alone as it promotes cell membrane damage and leads to the formation of oxidative radicals from matrix components (Giannakis, 2018). Additionally, enhanced H_2_O_2_ consumption during photo-Fenton reactions is associated with a higher generation of oxidative radicals (Eq. (1)), thus resulting in exposure of bacteria to highly hostile conditions. Exposition of bacteria to these conditions initially causes reduced damage, yet prolonged treatment times lead to accumulated injury and eventual cell death (Serna-Galvis et al., 2019; Verbel-Olarte et al., 2021).

In contrast, recent literature points out to the spread and selection of ARB and ARGs after the application of traditional technologies such as Chlorine, ozonation and UV-C irradiation/oxidant (Jin et al., 2020; Kirchner et al., 2020; Lee et al., 2021; Moreira et al., 2021; Sharma et al., 2019). In addition, ozonation and UV-C irradiated processes are energy intensive and costly, which may hinder their application in developing countries. Meanwhile, solar photo-Fenton explores a natural and costless energy source which is abundant in tropical developing countries.

Solar photo-Fenton using 30 mg L^−1^ of iron (intermittent additions) (Fig. S2b) had a higher impact upon microbial community diversity (640 OTUs) compared with 5 mg L^−1^ of iron (899 OTUs). Higher availability of Fe^2+^ in the system using 30 mg L^−1^ led to greater oxidant consumption (Fig. S2c) in the reaction between iron and H_2_O_2_ (Eq. (1)) and consequently higher generation of hydroxyl radicals (HO•) which react quickly with cell components such as DNA (10^8^–10^9^ M s^−1^) (Neta et al., 1982), thus promoting disinfection and decreasing the diversity of microbial community present in MWWTPE.

The intermittent iron addition strategy ensured the continuous presence of Fe^2+^ during reactions at neutral pH even after 60 min (Fig. S2c), thus being shown to be effectively overcoming the limitation associated with optimal pH for the operation of photo-Fenton (Carra et al., 2014, Carra et al., 2013; Clarizia et al., 2017; Díaz-Angulo et al., 2021). Fe^2+^ cycling (Eq. (2)) is enhanced in the photo-Fenton system, and an extra route for the formation of HO• (Eq. (3)) occurs under UV-A irradiation via light adsorption by iron hydroxides formed in the system (Tarr, 2003).

(1) $${{\mathrm{Fe}}_{2}}^{+}+H_{2}O_{2}\to {\mathrm{Fe}}^{3+}+\mathrm{HO}˙+{\mathrm{OH}}^{-}$$

(2) $${\mathrm{Fe}}^{3+}+H_{2}O_{2}\to {\mathrm{Fe}}^{2+}+{\mathrm{HO}}_{2}˙+H^{+}$$

(3) $$\mathrm{Fe}{\left(\mathrm{OH}\right)}^{2+}+{\mathrm{h\nu}}_{\left(540–580\;\mathrm{nm}\right)}\to {\mathrm{Fe}}^{2+}+\mathrm{HO}˙$$

**Fig. 1:**
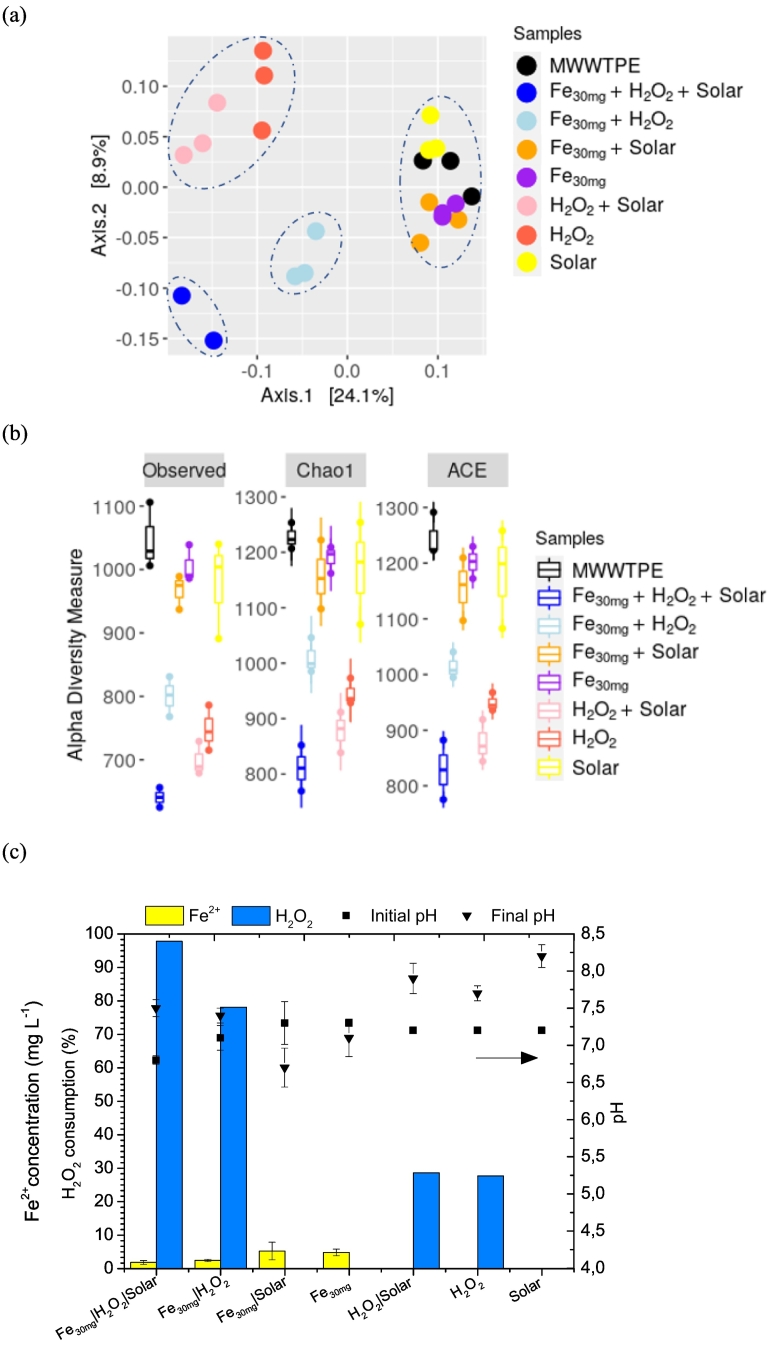
Beta diversity obtained by PCoA analysis (a), alpha diversity metrics obtained by non-parametric richness estimator (Chao 1 and ACE) (b), and pH, hydrogen peroxide consumption, and dissolved iron concentration after solar photo-Fenton treatment and controls carried out for 240 min (Q_uv_ = 22.28 kJ L^−1^) (c).

PCoA indicated significant (p < 0.05) differences in the taxonomic structure of bacteria community before and after treatment and controls (Fig. 1a). Solar photo-Fenton samples clustered on the bottom left side (Fig. 1a), showing significant differences in bacterial community diversity compared with MWWTPE samples (right upper side). The lowest average of microbiome diversity (Chao 1 diversity index) and abundance-based coverage estimator (ACE) (39% below MWWTPE sample) was detected in solar photo-Fenton samples, thus confirming the disinfection potential of this process (Fig. 1b). These results were confirmed by culture-based analysis of total heterotrophic bacteria and ARB. As shown in Fig. S2d, solar photo-Fenton treatment achieved nearly 70% removal of cultivable THB. The treatment was also efficient in eliminating ARBs (85% removal). ARB removal was enhanced under solar photo-Fenton (~1 log removal for ARB resistance to all tested antibiotics) compared to Fenton control for which removal of ARBs was limited to ~0.5 log, except for trimethoprim-resistant bacteria (~1 log removal).

Enhanced effect of solar photo-Fenton compared with control Fenton is associated with an increase in H_2_O_2_ consumption (95%) compared with control Fenton (Fig. 1c) (75%) due to the higher number of routes related to the formation of hydroxyl radical (Eqs. (1), (2), (3)), enhanced Fe^2+^ cycling and faster kinetics of HO• formation under irradiation (Malato et al., 2009). Different cell damaging mechanisms are responsible for the effect of solar photo-Fenton upon bacteria in MWWTPE, such as: (i) UVA alone increases cell permeability and promotes loss of Fe^2+^ from enzymes, thus launching an internal photo-Fenton process with the formation of reactive oxygen species (ROS) inside cells (Giannakis, 2018); (ii) secondary radicals formed by the exposition of natural organic matter and ions present in the matrix to UVA irradiation damage bacteria cell structure (Rommozzi et al., 2020); (iii) the UVB component of solar irradiation causes direct damage to DNA (Feng et al., 2020); (iv) external photo-Fenton reactions launched by the oxidative radicals formed in the bulk due to the addition of iron salts and H_2_O_2_ to the system intensify outer cell damage (Giannakis, 2018); and (v) transportation of H_2_O_2_ to the inner cell compartment via porins (Feng et al., 2020) enhances internal photo-Fenton reactions.

As shown in Fig. 1c, pH ranged from 6.5 and 7.5 during solar photo-Fenton and control Fenton. The pH stability during photo-Fenton at neutral pH using the intermittent iron addition strategy was also observed by Starling et al. (2021a). Recent studies indicate that solar photo-Fenton efficiency is hindered at neutral pH due to iron precipitation (Carra et al., 2013; Clarizia et al., 2017). However, the intermittent iron addition strategy mitigates this effect since dissolved iron is present in the system during the entire treatment. Iron precipitation occurs gradually, avoiding a turbidity peak usually associated with light scattering effects. Final Fe^2+^ concentration was under 5 mg L^−1^ after photo-Fenton and Fenton treatments, which is below discharge limits and presents no risks to aquatic environments (Silva et al., 2018; Starling et al., 2021a).

A cluster containing control samples (H_2_O_2_ + solar and H_2_O_2_) was formed on the upper left side (Fig. 1a). This is concurrent with results obtained in culture-based bacterial analyses of THB and ARB (Fig. S2d). These controls were less efficient in the removal of ARBs (except for ARBs resistant to tetracycline - removal ~1 log) compared to solar photo-Fenton treatment. Although H_2_O_2_ + solar control showed ~75% removal of THB (Fig. S2d), the control containing only H_2_O_2_ did not show a significant percentage of THB removal, being efficient only in removing tetracycline-resistant ARBs. The effect of these controls upon microbial community and cultivable bacteria may be associated with H_2_O_2_ consumption (nearly 30%) (Fig. 1c), which is related to the oxidation of matrix and bacteria cell components. H_2_O_2_ alone may disrupt the lipid bilayer of the bacteria cell membrane as it oxidizes lipids (Siddique et al., 2012). In addition, the transportation of H_2_O_2_ to the inner cell compartment and the release of Fe^2+^ from enzymes by the action of irradiation alone (Feng et al., 2020) promotes the internal photo-Fenton during H_2_O_2_ + solar (Giannakis, 2018), thus contributing to disinfection. Similar disinfection rates were also observed for solar photo-Fenton at neutral pH and H_2_O_2_ + solar in previously published articles (Giannakis, 2018; Maniakova et al., 2021; Michael-Kordatou et al., 2018; Michael et al., 2020). Although not assessed in this study, ARB regrowth may be used as an additional indicator of disinfection efficiency. Bacteria regrowth was observed after 48 h of storage following H_2_O_2_ + solar (Fiorentino et al., 2015; Michael et al., 2020) while no regrowth occurred after solar photo-Fenton treatment (Fiorentino et al., 2019).

The single cluster formed by MWWTPE and controls samples (Fe^2+^; solar; Fe^2+^ + solar) (right center side) (Fig. 1a), shows that these controls had no significant effect upon the original bacterial community. This is confirmed in alpha diversity analysis (Fig. 1b) as no significant difference was detected between control samples and MWWTPE. Lack of impact upon MWWTPE microbial community diversity after solar irradiation alone is consistent with reports made in other studies (Serna-Galvis et al., 2019; Verbel-Olarte et al., 2021).

### 3.3 Effect of solar photo-Fenton on bacterial phyla

**Fig. 2:**
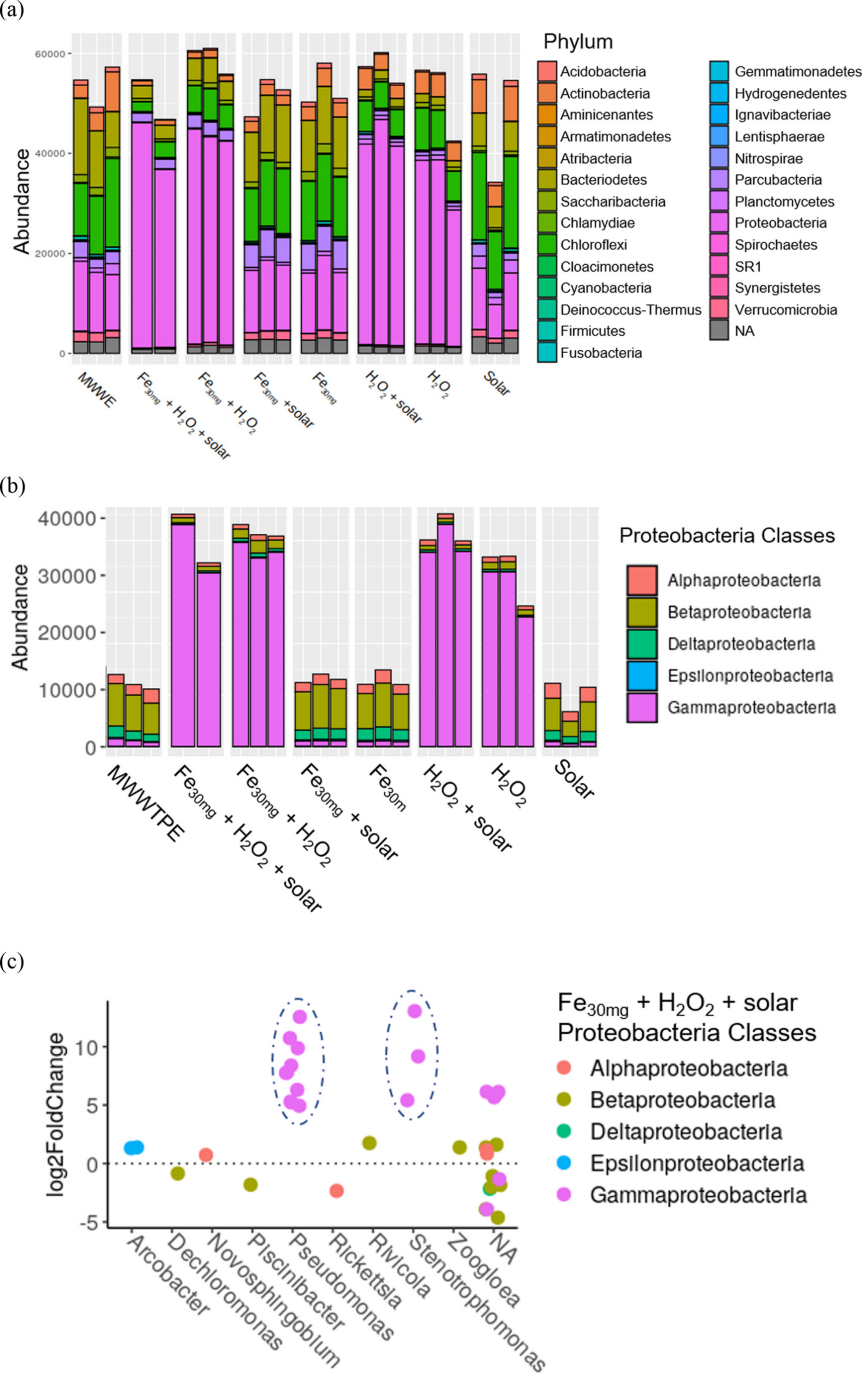
Phylogeny of bacterial communities (a); abundance of *Proteobacteria* classes (b); and classes and genera of *Proteobacteria* in samples treated by solar photo-Fenton (treatment time: 240 min, Q_uv_ = 22.28 kJ L^−1^) (c). Taxa with abundance below 1% and unclassified taxa were designated as NA.

Solar photo-Fenton effectively removed most of the main phyla present in MWWTPE (Fig. 2a), achieving 86% removal of *Acidobacteria*, 80% removal of *Chloroflexi*, and around 79%, 76%, and 74% removal of *Actinobacteria*, *Bacteriodetes*, and *Firmicutes*, respectively. Unclassified taxa were reduced by 67%. These results are promising since priority ARB belong to *Proteobacteria* (e.g., *P. aeruginosa*, *A. baumannii*, *N. gonorrhea*, etc.), *Firmicutes* (e.g., *E. Faecium*, *S. pneumoniae*, *S. aureus*), and *Bacteroidetes* (e.g., *C. normanense*, *C. meningosepticum*) phyla (Quintela-Baluja et al., 2019; Su et al., 2017). Besides, bacteria from *Proteobacteria* and *Actinobacteria* phyla are major hosts of ARGs carried by Methicillin-Resistant *Staphylococcus aureus* [MRSA]-related (*mec*A, *qac*A, *qac*B, *nor*A) and carbapenem-Resistant *Enterobacteriaceae* [CRE] (KPC, NDM, OXA-48) (Yin et al., 2019; Zhang et al., 2021, Zhang et al., 2019).

An almost four-fold enrichment was observed for *Proteobacteria* in all samples containing H_2_O_2_, thus indicating a positive selection of this group (Fig. 2a). *Proteobacteria* selection was also observed after ozonation of municipal wastewater (Moreira et al., 2021) and suggested higher resistance of this group to oxidative conditions. The bacterial community present in MWWTPE is sensitive to H_2_O_2_, which can function either as a disinfectant (Apel and Hirt, 2004) or as an oxygen source enhancing aerobic bacterial growth (Hinchee et al., 1991; Zappi et al., 2000). Treated samples presented five major classes within *Proteobacteria* phylum (Fig. 2b). Among these classes, the highest increase in solar photo-Fenton samples was observed for *Gammaproteobacteria*, mainly within genera *Pseudomonas* and *Stenotrophomonas* (Fig. 2c). Relative abundance of *Gammaproteobacteria* class also increased in the presence of H_2_O_2_ and after solar-driven AOPs elsewhere (Moreira et al., 2018; Wang et al., 2017). Selection of *Pseudomonas* genera during solar photo-Fenton may be associated with their tolerance to H_2_O_2_ (Bjarnsholt et al., 2005; Moreira et al., 2018) as they possess mechanisms to eliminate H_2_O_2_ present in the environment (Magro et al., 2019). H_2_O_2_ scavenging mechanisms were associated with eight genes (KatA1, KatA2, KatMn, and KatE, AhpCF, Gpx1, Gpx2, and Gpx3) in *Stenotrophomonas* sp. Among these genes, KatA2 played a critical role for survival in the presence of high H_2_O_2_concentrations (2.0 mM) (Li et al., 2020). Solar photo-Fenton and control Fenton samples presented a 4–5 fold growth in the KatA2 and KatE genes, and AhpCF increased by 33% (data not shown). Thus, suggesting a significant adaptive response to H_2_O_2_ stress (data not shown). Besides, *Pseudomonas* is known for carrying genes that encode efflux pumps, thus conferring resistance to both antibiotics and other disinfecting agents (Pang et al., 2019). *Pseudomonas* may also have benefited from the oxidation of organic components present MWWTPE via solar photo-Fenton as they have the ability to degrade carbonyl compounds as a feeding source (Johnson et al., 2011).

Critical priority of *Pseudomonas aeruginosa* (WHO, 2017) and *Stenotrophomonas maltophilia*, an emerging pathogen responsible for high morbidity and mortality (Gil-Gil et al., 2020), were both detected in MWWTPE samples (data not shown). Solar photo-Fenton reached total elimination of these species, while control Fenton eliminated almost 30% of *Stenotrophomonas maltophilia* and full removal of *Pseudomonas aeruginosa*. This confirms the potential of solar photo-Fenton to eliminate priority pathogens from MWWTPE, thus contributing to public health improvement.

#### 3.3.1 Effect of solar photo-Fenton on priority pathogens

**Fig. 3:**
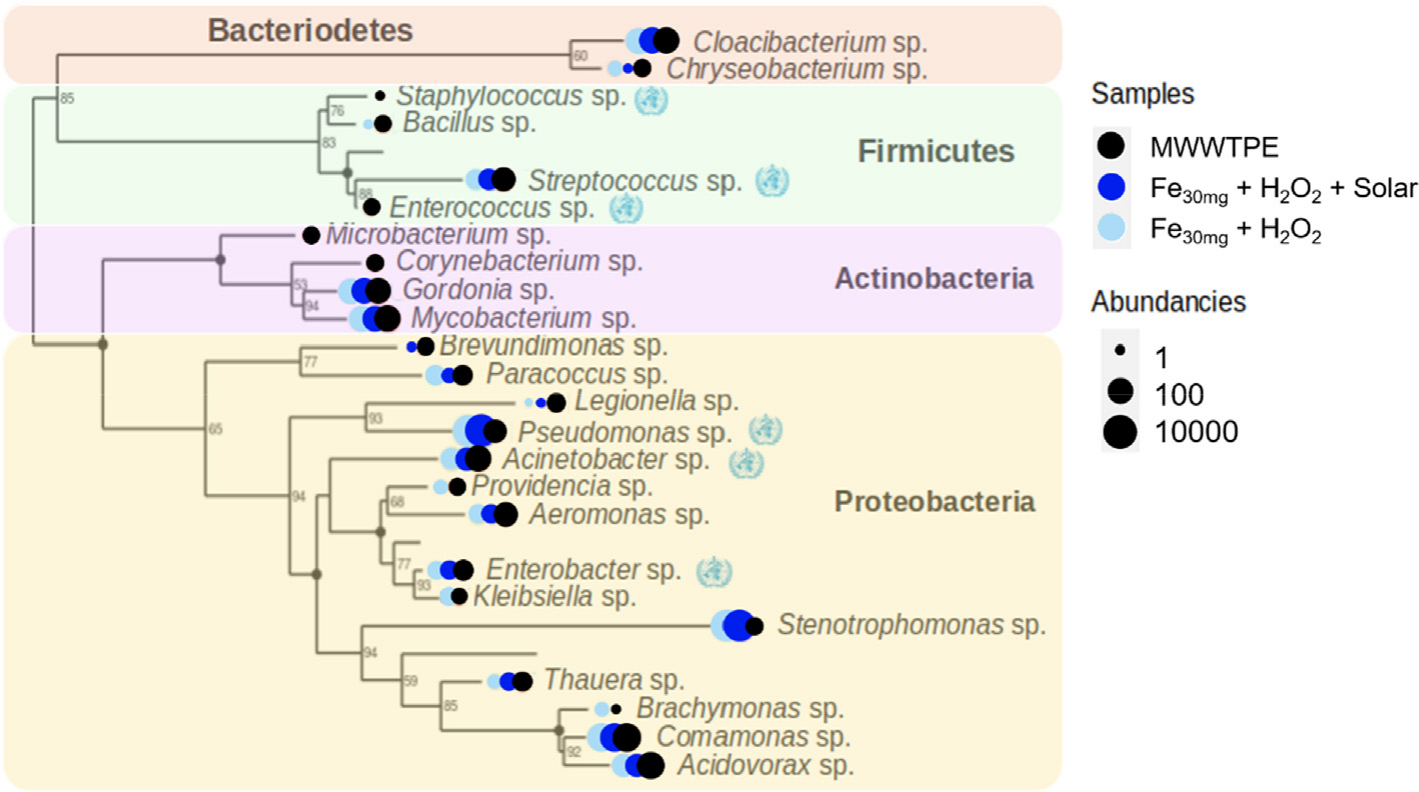
Phylogenetic tree showing the occurrence of WHO priority pathogens and selected genera in MWWTPE and samples treated by solar photo-Fenton and control Fenton (treatment time: 240 min, Q_uv_ = 22.28 kJ L^−1^). The blue symbol corresponds to the WHO logo added to represent each of the priority pathogens.

Six potential pathogens belonging to critical, high, and medium priority classes were detected in original MWWTPE samples (WHO, 2017) (Fig. 3). Solar photo-Fenton and Fenton completely removed high priority *Staphylococcus* and *Enterococcus* (Fig. 3). *Staphylococcus* infections carry high mortality levels when aggravated by antimicrobial resistance (Giulieri et al., 2020), and *Enterococcus* is an opportunistic pathogen associated with increased mortality rates (Leavis et al., 2006). In contrast, *Streptococcus* sp., inserted in the same group, was not entirely removed by proposed treatments. Failure to eliminate this priority group may be associated with its ability to adapt membrane composition in hostile oxidative environments (Pesakhov et al., 2007).

Regarding *Acinetobacter* genera, phylogeny analysis of solar photo-Fenton samples revealed an absence of *A. baumannii*, a critical priority pathogen (WHO, 2017). *A. johnsonii* predominated in all samples. Despite rarely causing human infections, this organism may actively acquire exogenous DNA becoming an ARG reservoir (Montaña et al., 2016). In the *Enterobacteriaceae* family, Fenton was not efficient at removing *Escherichia coli* or *Klebsiella pneumoniae* (*Enterobacteriaceae* family). Nevertheless, solar photo-Fenton completely removed *Klebsiella pneumoniae* and achieved 30% removal of *E. coli*. *Klebsiella pneumoniae* is known for having a negatively charged outer capsule (Podschun and Ullmann, 1998) which may have complexed with iron, thus lowering Fenton efficiency in the absence of light. Elimination of *Klebsiella pneumoniae* from MWWTPE is critical to limit the spread of AMR as it is a major cause of hospital and community-acquired infections (Munoz-Price et al., 2013).

Growth of *Pseudomonas* and *Stenotrophomonas* was associated with their tolerance to H_2_O_2_. Regarding species within the *Pseudomonas* family, *P. yamanorum* predominated in treated samples. Within *Stenotrophomonas*, *S. maltophilia*, a harmful β-lactam resistant pathogen (Kumar et al., 2020), and *S. pavanii* ruled in MWWTPE sample, yet were entirely removed by solar photo-Fenton.

Regarding bacteria known as ARG vectors, solar photo-Fenton achieved a substantial reduction of intrinsically multi-drug resistant *Chryseobacterium*. This is relevant as this genera is known for its resistance to chlorination applied in conventional MWWTP (Izaguirre-Anariba and Sivapalan, 2020). Solar photo-Fenton and Fenton achieved significant removal of *Legionella* and *Brevundimonas* (*Proteobacteria* cladon), commonly associated with nosocomial infections and considered pathogens of emerging concern in clinical locations (Li et al., 2018; Lytle et al., 2021; Ryan and Pembroke, 2018). In contrast, *Gordonia* sp., an opportunistic agent (Blaschke et al., 2007), and *Mycobacterium* sp. (*Corynebacteriales*; *Actinobacteria*), were not removed after Fenton and solar photo-Fenton. Extensively TB drug-resistant and multidrug-resistant organism *Mycobacterium tuberculosis* (Dua et al., 2018; Kumar et al., 2020) was not detected in any of the samples in this study (data not shown).

### 3.4 Effect of solar photo-Fenton on resistome profile: diversity and richness of ARGs

**Fig. 4:**
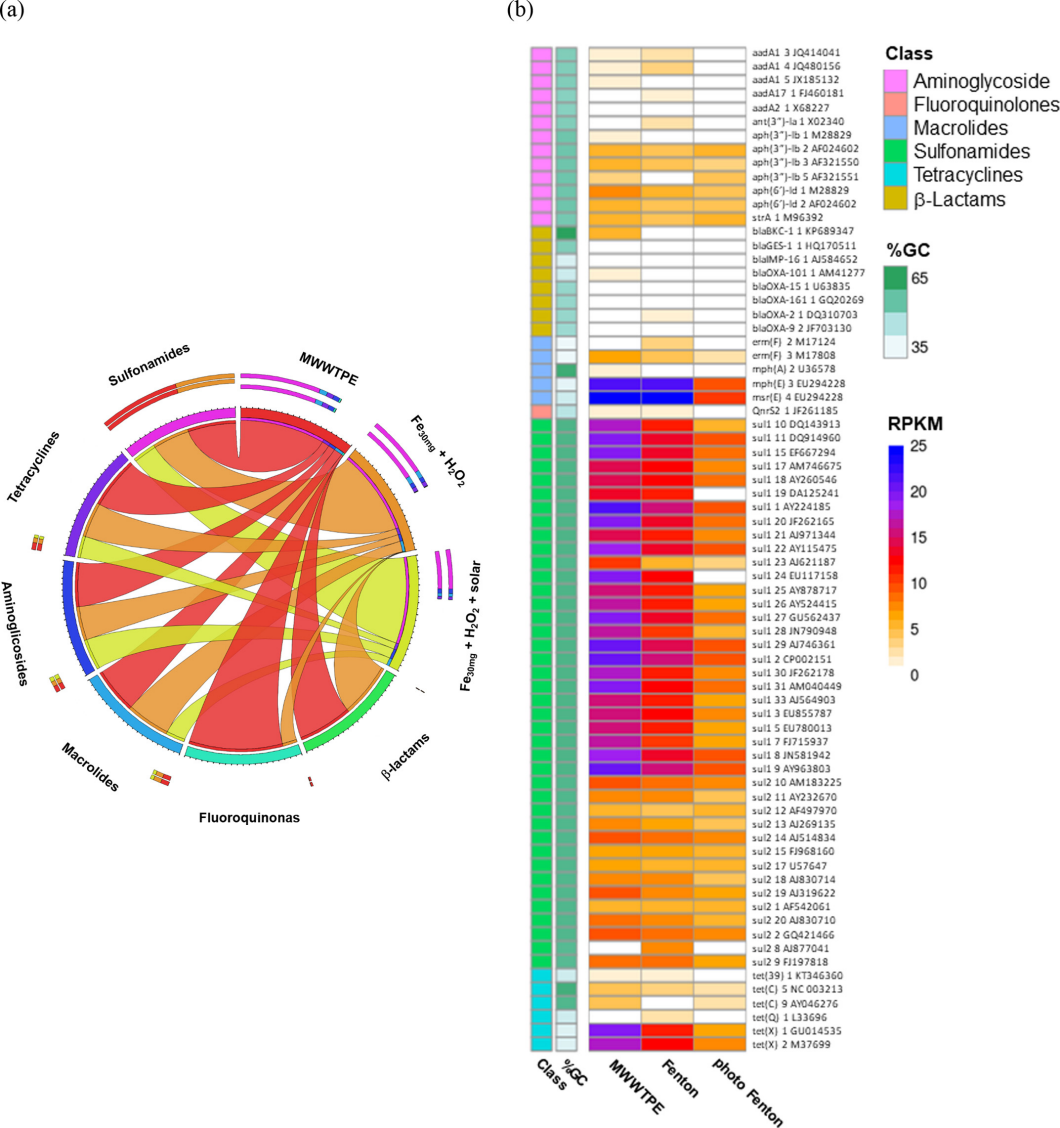
Relative occurrence of ARGs (a); Heatmap of the distribution of ARGs as a percentage of reads per kilobase per million mapped reads (RPKM) in MWWTPE and samples obtained after solar photo-Fenton, and control Fenton (treatment time: 240 min, Q_uv_ = 22.28 kJ L^−1^) (b).

According to Fig. 4a, ARGs which confer resistance to different classes of antibiotics are abundant in the MWWTPE used in this study. Most current studies associated with ARG removal via solar photo-Fenton investigate a limited list of ARGs (bla_CTX_, bla_TEM_, bla_OXA_, sul1, sul2, emrB, tetQ, tetX, and tetM) via qPCR (Starling et al., 2021b). This is the first study to use WGS to investigate ARG removal from MWWTPE. A greater ARGs diversity (69 variations within 19 major types) (Fig. 4b) was detected, and their removal was analyzed in this study.

ARGs which confer resistance to broad-spectrum antibiotics, such as sulfonamides (sul1 and sul2), represented almost 74% of total ARGs present in MWWTPE, followed by ARGs associated to macrolides (mainly erm(F), mph(A), mph(E) and msr(E)) (~10%), tetracyclines (mainly tetA, tetB, tetO and tetW) (~9%) and aminoglycosides (mainly aadA, aph(3″), aph(6) and strA) (~6%). Overall, subtypes tet(X)_1, tet(X)_2, mph(E)_3 and msr(E)_4, and genes sul1 and sul2 (almost all subtypes) were the most abundant across MWWTPE samples (Fig. 4b). According to Nguyen et al. (2021) and Raza et al. (2021), ARGs conferring resistance to sulfonamides and tetracyclines are frequently detected in MWWTPE regardless of predominant bacteria taxa, season, and location. Gene sul1 usually prevails in MWW worldwide due to high sequence conservation and transfer among different species (Wei et al., 2018).

Solar photo-Fenton removed nearly 60% of ARGs associated with sulfonamides (55%), macrolides (61%), and tetracyclines (61%), and wholly removed ARGs associated with β-lactams and fluoroquinolones. Regarding subtypes, the treatment removed 66% of tet(X)_1 and almost 60% of tet(X)_2, mph(E)_3, and msr(E)_4 (Fig. 4b). Complete removal of ARGs associated with β-lactams is of remarkable relevance. Some of them are emerging threats to public health (i.e., carbapenem-resistant *Enterobacteriaceae*-related genes: BKC, GES, IMP, OXA, etc.) (Bush and Jacoby, 2010; Logan and Weinstein, 2017). Effect of control Fenton upon ARGs was limited to 24% and 30% for ARGs associated with sulfonamides and tetracyclines, respectively, and no substantial decay of ARGs associated to macrolide (~3%) and fluoroquinolone (~7%) classes were detected after treatment.

Results obtained by the most recent works published on the application of solar photo-Fenton have shown high efficiency of ARGs removal. Michael et al. (2019) reached complete removal of sul1, qnrS, blaOXA, blaCTX-M, and tetM, and 3 log units of blaTEM removal and Fiorentino et al. (2019) removed sul1 genes to levels below the detection limits. While solar photo-Fenton successfully removed ARGs (CTX-M-1 and CTX-M-9), sunlight/H_2_O_2_ failed to remove these genes (Giannakis, 2018). These results agree with high efficiency of ARG removal obtained in our study.

Limited removal of ARGs related to aminoglycosides via solar photo-Fenton (36% removal) and control Fenton (~26% removal) may be associated with the low GC content (~45%) of these genes (Fig. 4b). Oxygen species usually react more readily with guanine bases in the DNA. Thus lower degradation rates are expected for genes presenting reduced GC content (Ren et al., 2018; Zhang et al., 2016). Removal rates above 50% were detected for sul1, sul2, and tet(C), and mph(A) and blaBKC were completely removed via solar photo-Fenton due to high GC content (~65%) associated with these ARGs. Despite having low GC content (~35%), tet(X)_1, tet(X)_2, mph(E)_3, and msr(E)_4 were significantly removed by solar photo-Fenton (>60%) as they were abundant in MWWTPE. The persistence of sulfonamide and tetracycline genes after proposed oxidative treatment is associated with their high initial concentration in MWWTPE. Therefore, these ARGs may be potential indicators of ARG removal from MWWTPE.

### 3.5 Correlation between bacterial community and antibiotic resistance genes

**Fig. 5:**
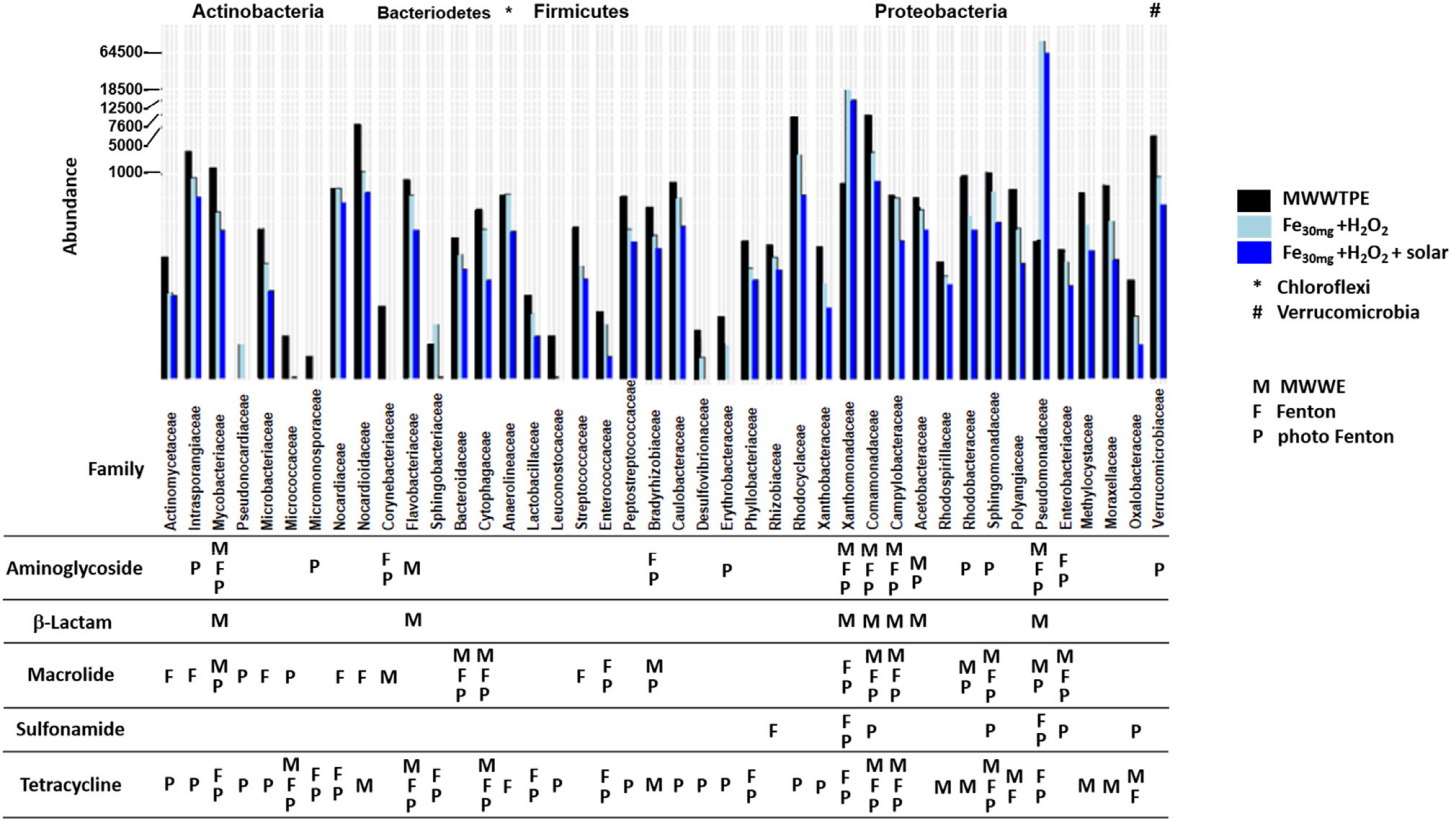
Correlation between the absolute abundance of specific phyla and family levels carrying ARGs and the ARGs subtypes present in MWWTPE and samples treated by control Fenton and solar photo-Fenton (treatment time: 240 min, Q_uv_ = 22.28 kJ L^−1^).

As co-existing patterns between ARGs and bacterial communities indicate potential hosts of AMR (Jia et al., 2020) and bacterial community is considered one of the main drivers of ARGs spread, specific relationships between bacterial hosts and ARG subtypes were investigated for MWWTPE used in this study before and after solar photo-Fenton treatment. Taxa of contigs carrying ARGs were predicted at their specific phyla and family levels, respectively (Fig. 5). As shown in Fig. 5, different ARG-taxa relations were observed before and after solar photo-Fenton and control Fenton treatments. For example, tetracycline and macrolide resistance genes were correlated with almost all families in MWWTPE, while sulfonamide, β-Lactam, and aminoglycoside resistance genes correlated mostly with *Proteobacteria* (*Pseudomonadaceae* and *Xanthommonadaceae*).

Although solar photo-Fenton treatment decreased the richness and diversity of the bacterial community in MWWTPE (Fig. 1a and b), significant enrichment in the abundance of *Proteobacteria* was observed after treatment (Fig. 2a). *Proteobacteria* have been reported to be potential hosts of genes that carry resistance to aminoglycosides (i.e., strA, aph, and aadA) and tetracyclines (i.e., tetQ, tetC, and tetX) (Luo et al., 2021). Therefore, the increased abundance of this phylum contributed to the persistence of these ARGs after treatment (Fig. 5). Within *Proteobacteria*, *Pseudomonadaceae*, *Enterobacteriaceae*, *Campylobacteraceae*, *Comamonadaceae*, and *Xanthommonadaceae* also showed a strong correlation with ARGs related to sulfonamides and macrolides in MWWTPE samples. Increased abundance of *Pseudomonadaceae* and *Xanthommonadaceae* after treatment justifies the persistence of these ARGs after control Fenton and solar photo-Fenton.

The co-existence of ARGs associated with tetracycline, macrolides, and aminoglycosides (Fig. 5) was correlated with almost all families of bacteria present in MWWTPE. These ARGs persisted after treatment even after high removal rates (>60%) since they were abundant in the untreated sample (Fig. 4b). Thus, relatively low removal of aph(3″), aph(6″), strA, mph(E), msr(E), and tetX (Fig. 4b) might be due to their occurrence in a varied spectrum of hosts.

Notably, families comprising multidrug-resistant bacteria (*Mycobacteriaceae*, *Flavobacteriaceae*, *Xanthommonadaceae*, *Campylobacteraceae*, *Sphingomonadaceae*, *Pseudomonadaceae*, and *Enterobacteriaceae*) were the leading carriers of beta-lactam resistance genes in MWWTPE samples (Fig. 5). Nevertheless, these ARGs were removed after the proposed treatment (Fig. 5), confirming the results shown in Fig.4.

Results obtained here confirm the elimination of priority pathogens (Fig. 3) and ARGs (Fig. 4, Fig. 5) via solar photo-Fenton, thus ensuring the combat of AMR spread via MWWTPE discharge by this process. Nonetheless, some groups known as co-hosts of ARGs were selected during treatment. This fact requires further investigation and points out the use of these groups as potential AMR indicators. The establishment of global and regional indicators of AMR is critical for the control of priority pathogens and has been currently under discussion by the scientific community (Di Cesare et al., 2020).

## 4 Conclusions

The evaluation of the effects of solar photo-Fenton upon bacterial communities, priority pathogens, and ARGs using metagenomic analyses presented in this study appear to be novel in the scientific literature. The lowest species richness and diversity were achieved via solar photo-Fenton (30 mg L^−1^ of Fe^2+^ and 50 mg L^−1^ of H_2_O_2_; 240 min) compared to controls as the intermittent iron addition strategy was effective for treatment conduction at neutral pH. Solar photo-Fenton effectively removed the main phyla present in MWWTPE (86% removal of *Acidobacteria*, 80% of *Chloroflexi*, 79% of *Actinobacteria*, 76% of *Bacteriodetes*, and 74% of *Firmicutes*). Solar + H_2_O_2_ and H_2_O_2_ alone showed a lower impact upon the microbial community when compared to solar photo-Fenton. Enrichment of *Proteobacteria* after the application of the solar oxidative treatment should be further investigated as it indicates positive selective pressure and led to the persistence of ARGs carried by this group in treated samples. Complete removal of high priority *Staphylococcus* and *Enterococcus*, critical priority *K. pneumoniae* and *P. aeruginosa*, and *S. maltophilia*, as well as substantial reduction of multi-drug resistant bacteria were observed. The proposed treatment also reached nearly 60% removal of ARGs associated with sulfonamides, macrolides, and tetracyclines, as well as complete removal of those related to β-lactams and fluoroquinolones. These results confirm the potential of applying solar photo-Fenton as an additional treatment stage in MWWTP to control the spread of AMR in tropical countries.

## Funding sources

This work was supported, in the whole, or part, by the 10.13039/100000865Bill & Melinda Gates Foundation, Seattle, WA [Grand Challenges Exploitations Brazil, grant number OPP1193112] under the grant conditions of the Foundation, a Creative Commons Attribution 4.0 Generic License has already been assigned to the Author Accepted Manuscript version that might arise from this submission. The 10.13039/501100004901Foundation for Research Support of the state of Minas Gerais (FAPEMIG); 10.13039/501100002322Coordination for the Improvement of Higher Education Personnel (CAPES); and the 10.13039/501100003593National Council for Scientific and Technological Development (CNPq) have also supported this work.

## CRediT authorship contribution statement

Study conception and design – P. B. Vilela, Rondon P. Mendonça Neto, A. S. Martins, M. C. V. M. Starling, and C. C. Amorim; experimental data - P. B. Vilela, Rondon P. Mendonça Neto, Felipe A. R. de Souza, Pires, G. F. F., A. S. Martins; manuscript writing and revision - P. B. Vilela, Rondon P. Mendonça Neto, M. C. V. M. Starling, and C. C. Amorim. All authors read and approved the final manuscript.

## Declaration of competing interest

The authors declare that they have no known competing financial interests or personal relationships that could have appeared to influence the work reported in this paper.

## Appendix A Supplementary material

Supplementary material Image 1
