# Supplementary material for: Metagenomic analysis of MWWTP effluent treated via solar photo-Fenton at neutral pH: Effects upon microbial community, priority pathogens, and antibiotic resistance genes
Source: Sci Total Environ. 2021 Dec 20;801:149599. doi: 10.1016/j.scitotenv.2021.149599 (PMC8573595; doi:10.1016/j.scitotenv.2021.149599)
Supplement: Supplementary file 1 — Supplementary material [file mmc1.docx]

Supporting Information

Metagenomic analysis of MWWTP effluent treated via solar photo-Fenton at neutral pH: Effects upon microbial community, priority pathogens and antibiotic resistant genes

Pâmela B. Vilela^a^, Rondon P. Mendonça Neto^a,b^, Maria Clara V. M. Starling^a^, Alessandra da S. Martins^a^, Giovanna F. F. Pires^a^, Felipe A. R. Souza^a^, Camila C. Amorim^a*^

^a^ Universidade Federal de Minas Gerais, Escola de Engenharia, Departamento de Engenharia Sanitária e Ambiental, Research Group on the Environmental Application of Advanced Oxidation Processes (GruPOA), Av. Pres. Antônio Carlos, 6627, 31270-901, Belo Horizonte - MG, Brazil,

^b^ Universidade Federal de Minas Gerais, Instituto de Ciências Biológicas, Departamento de Bioquímica e Imunologia, Pampulha, Belo Horizonte - MG, Brazil

^*^Corresponding author: Camila C. Amorim, e-mail: camila@desa.ufmg.br

Table S1 - Physicochemical characterization (median values) of MWWTPE (n = 11) considering main wastewater quality parameters: Chemical Oxygen Demand (COD), pH, Turbidity, Total Solids (TS), Volatile Solids (VS), Fixed Solids (FS), Alkalinity, and Conductivity as according to APHA (2017). Total Organic Carbon (TOC), Total Inorganic Carbon (TIC) and Total Nitrogen (TN) were analyzed by a Total Organic Carbon Analyzer (Shimadzu TOC-V CPN).

| Parameter | | x̅ ± σ | Reference |
| --- | --- | --- | --- |
| COD | mgO_2_ L^-1^ | 72 ± 32 | APHA 5220 D |
| pH | - | 7.2 ± 0.2 |  |
| Temp. | °C | 24 ± 0.6 |  |
| TOC | mg L^-1^ | 10.33 ± 7.4 |  |
| CT | mg L^-1^ | 53.02 ± 15.1 |  |
| TIC | mg L^-1^ | 28.45 ± 18 |  |
| TN | mg L^-1^ | 37.23 ± 18.9 |  |
| Turbidity | NTU | 24.3 ± 19.1 |  |
| TS | mg L^-1^ | 347 ± 176 | APHA 2540 B |
| VS | mg L^-1^ | 156 ± 80 | APHA 2540 E |
| FS | mg L^-1^ | 196 ± 97 | APHA 2540 C |
| Alkalinity | mgCaCO_3_ L^-1^ | 157 ± 47 | APHA 2320 B |
| Conductivity | µS cm^-1^ | 461 ± 167 | APHA 2510 B |

Fig. S1 – Relative abundance of bacterial phyla in MWWTPE samples (*n* = 11) during wet and dry seasons. Taxa with an abundance below 1% and unclassified taxa were designated as NA.


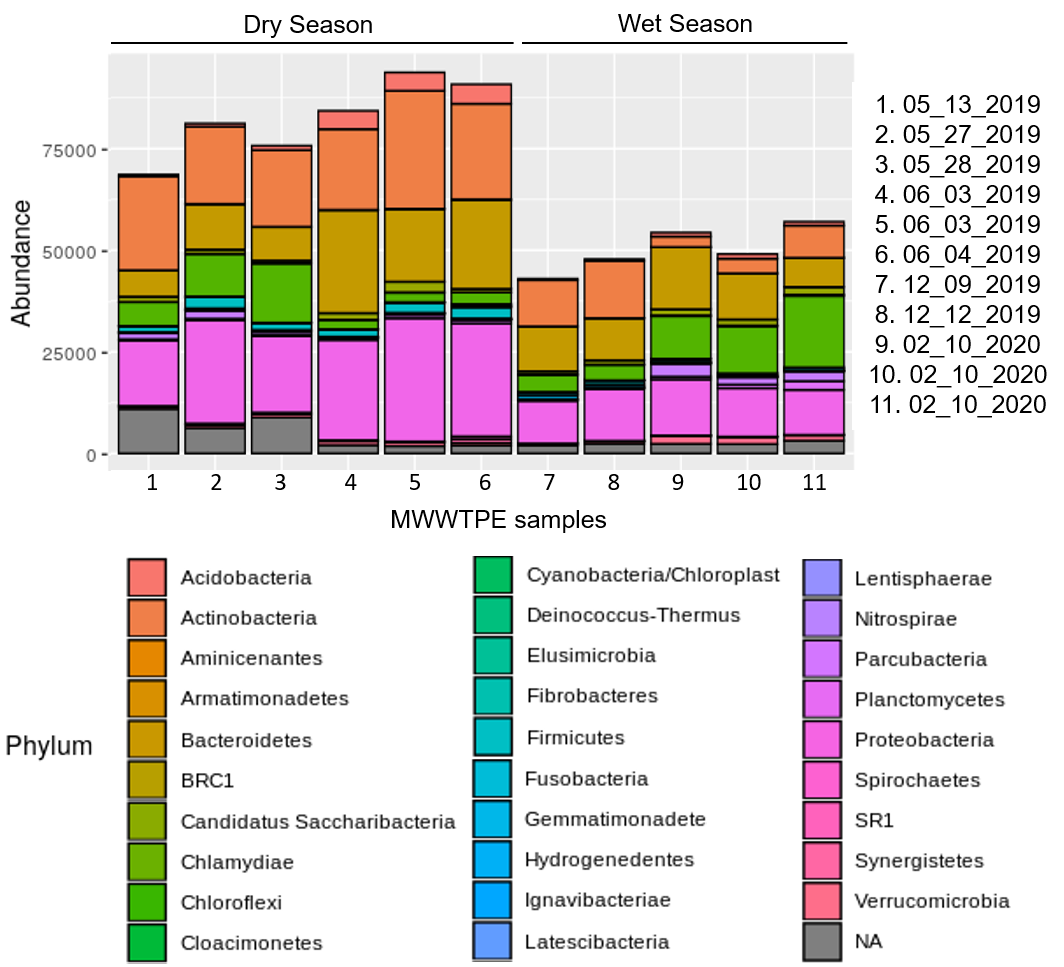


Fig. S2. Principal coordinates analysis (PCoA) based on Bray-Curtis dissimilarity index among the samples of MWWTPE, solar photo-Fenton, and Fenton conducted for 120 or 240 minutes (a), comparison of alpha diversity metrics for solar photo-Fenton and control treatments using 30 or 5 mg L^-1^ of Fe^2+^ (MWWTPE = 1047 OTUs; Solar photo-Fenton 30 mg L^-1^ = 640 OTUs; solar photo-Fenton 5 mg L^-1^ 898 OTUs; control Fenton mg L^-1^ = 980 and 30 mg L^-1^ of Fe^2+^ = 800 OTUs). (b) Dissolved iron concentration and hydrogen peroxide consumption (c), and log removal of total heterotrophic bacteria and ARB (d).

| (a) |
| --- |
| 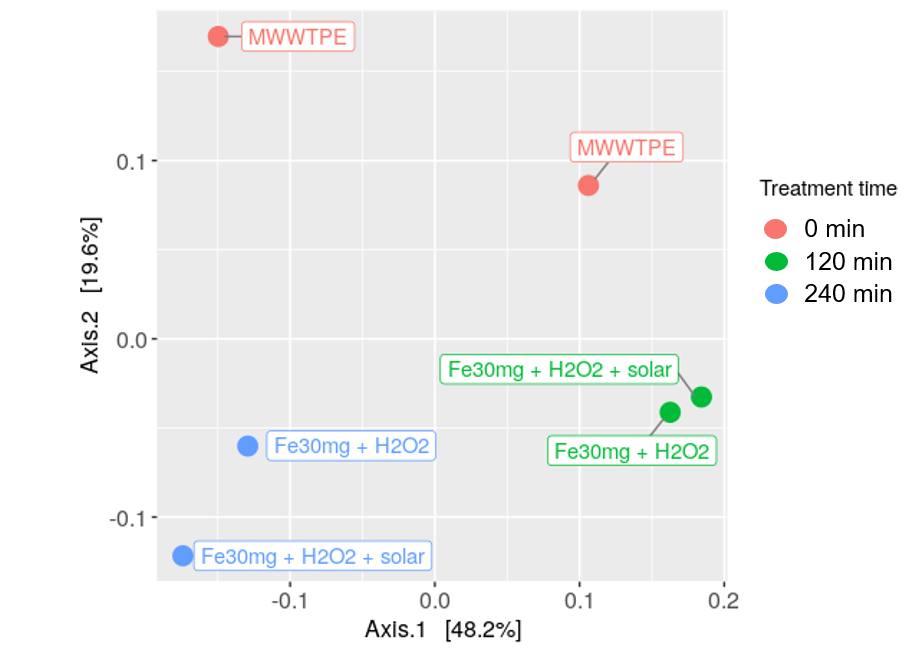 |

| (b) |
| --- |
| 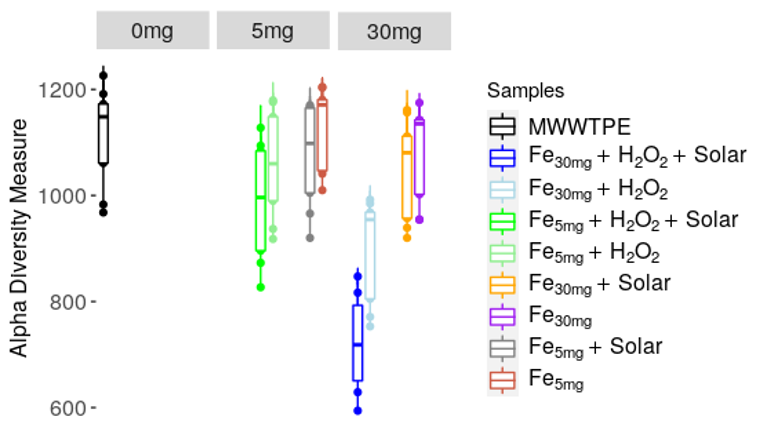 |
| (c) |
|  |
| (d) |
|  |

**Reference**

APHA. 2017. Standard Methods for the Examination of Water and Wastewater. 23 ed. Washington: APHA.
